# In Vitro Investigation of Microcatheter Behavior During Microsphere Injection in Transarterial Radioembolization

**DOI:** 10.1177/15266028251318953

**Published:** 2025-02-24

**Authors:** Tess Josien Snoeijink, Jan Lucas van der Hoek, Hadi Mirgolbabaee, Tristan Gerard Vlogman, Joey Roosen, Johannes Frank Wilhelmus Nijsen, Erik Groot Jebbink

**Affiliations:** 1 Department of Medical Imaging, Radboud University Medical Center, Nijmegen, The Netherlands; 2 Multi-Modality Medical Imaging Group, TechMed Centre, University of Twente, Enschede, The Netherlands; 3 Physics of Fluids Group, TechMed Centre, University of Twente, Enschede, The Netherlands; 4 Department of Thermal and Fluid Engineering, Faculty of Engineering Technology, University of Twente, Enschede, The Netherlands

**Keywords:** radioembolization, TARE, microspheres, distribution, in vitro

## Abstract

**Purpose:**

To experimentally investigate the behavior of a clinically used microcatheter during transarterial radioembolization (TARE) microsphere injection in a successively bifurcating *in vitro* model.

**Materials and Methods:**

A symmetrical phantom was developed which bifurcated 3 times into 8 outlets. A blood-mimicking fluid was pumped through the phantom using a physiological representative waveform. Holmium-165 microspheres were injected in a pulsed manner at 3 different locations using a standard microcatheter and a rigid counterpart with same dimensions as a control. Motion of the catheter was studied with a top- and side-view camera on the phantom. Microspheres were collected at each outlet and their distribution over the 8 outlets was analyzed.

**Results:**

Due to the pulsatile flow in the phantom, strengthened by the pulsatile microsphere injection, the clinical catheter showed maximum displacements of 0.87 mm within a vessel with a diameter of 3.6 mm. This motion resulted in a different microsphere distribution for the clinical catheter compared with the rigid counterpart (75.9% vs 49.4% of the microspheres went to outlet 1–4, respectively).

**Conclusion:**

In this *in vitro* model, the motion of the clinical catheter affected distribution of microspheres. Since the pulsatile administration of microspheres resulted in increased motion of the clinical catheter, standardizing microsphere administration could be beneficial to reduce interprocedural differences in TARE.

**Clinical Impact:**

Our study demonstrated that microsphere distribution during transarterial radioembolization (TARE) is affected by catheter motion. Furthermore, increased catheter motion was observed as a result of the injection profile. Predictive tools such as the contrast CBCT and scout dose use different injection profiles compared to therapeutic TARE injections, potentially altering catheter tip behaviour and microsphere distribution, which could compromise their predictive values. Additionally, current TARE microsphere injection guidelines provide limited details, which may lead to variability across institutes and interventional radiologists. Standardizing injection techniques could reduce catheter motion variability and may facilitate more consistent and predictable microsphere distribution patterns.

## Introduction

Transarterial radioembolization (TARE) is an established treatment method for primary and secondary liver cancer,^1,2^ involving a catheter-based intraarterial injection of radioactive microspheres. The treatment relies on the fact that liver tumors are predominantly supplied by the hepatic artery, while healthy liver parenchyma receives most of its blood supply from the portal vein.^3,4^ Microspheres are transported through the arterial liver vasculature until they lodge predominantly in and around the tumor to deliver a high local radiation dose.^5^

Trials such as the DOSISPHERE-01 and the subgroup analysis of the SARAH patient cohort showed improved survival and disease control for higher tumor doses.^6,7^ To enhance tumor to nontumor (T/N) ratios, more control over the distribution of microspheres is crucial. If fewer microspheres lodge in healthy liver tissue, higher radiation doses can be administered to the tumor.

To further optimize microsphere distribution during TARE, axial and radial (ie, cross-sectional) catheter tip position is a commonly investigated parameter, using *in vitro* and *in silico* (computer) models.^8–10^ *In vitro*, van den Hoven et al^10^ demonstrated homogeneous distributions with a centrally positioned catheter tip and skewed distributions with an off-centered tip in a surrogate model of human hepatic geometry. Although catheter tip position is widely studied, the influence of motion of the highly flexible catheter tip inside the vessel has not been investigated. It might be that under the influence of the pulsatile blood flow or injection, the radial position of the flexible catheter tip varies during microsphere injection.

The aim of this study was to investigate the behavior of a standard end-hole microcatheter during microsphere injection in a successively bifurcating phantom. A comparison between a clinical microcatheter and a rigid counterpart aims to unravel differences in microsphere distribution attributable to the flexibility and stability of the catheter tip.

## Materials and Methods

### Experimental Setup

**Figure 1.**
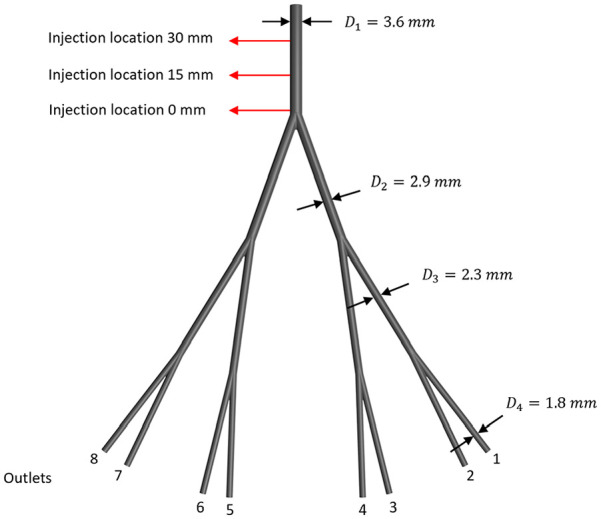
Successively bifurcating planar phantom. The *in vitro* model features a parent vessel with a diameter similar to the diameter of the right hepatic artery and bifurcates symmetrically 3 times into 8 outlets. Injection locations are indicated by the 3 red arrows; 0, 15, and 30 mm from the first bifurcation.

**Figure 2.**
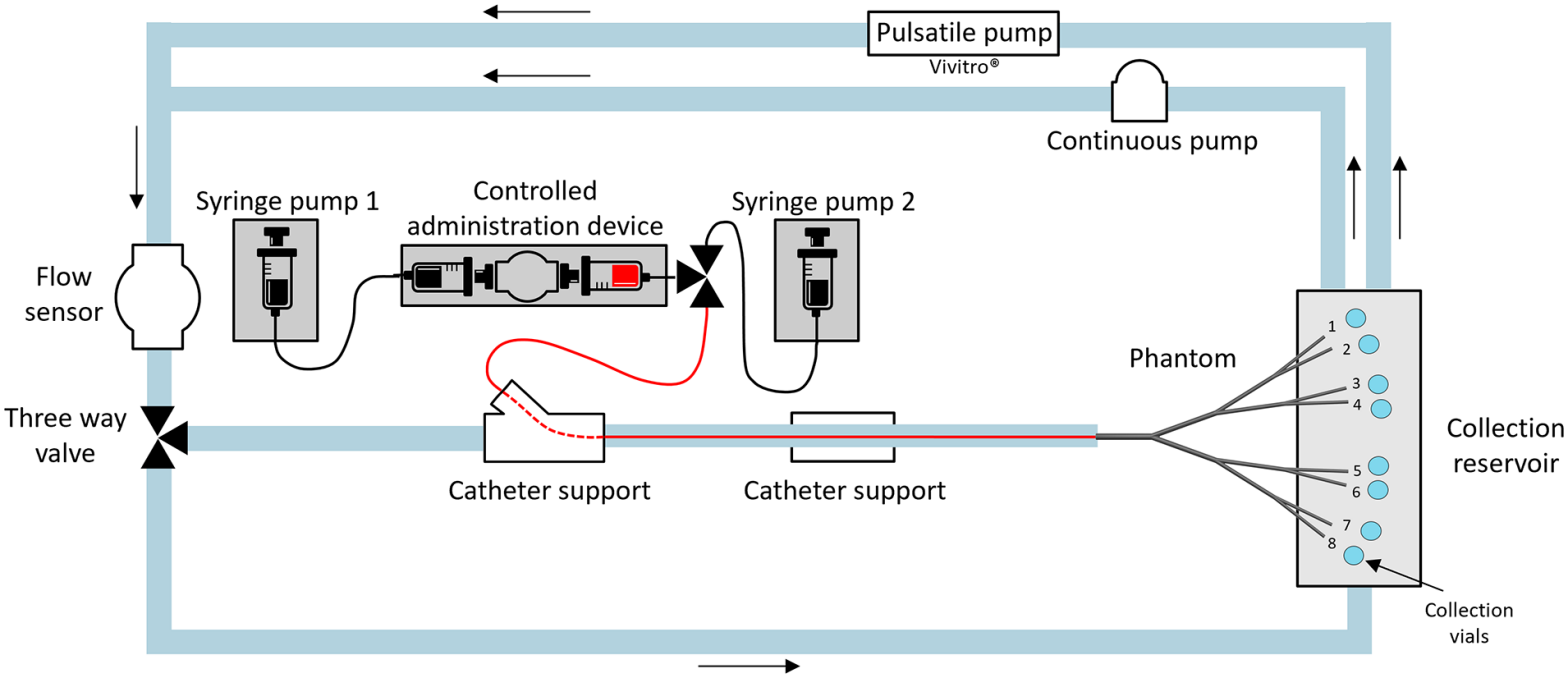
Schematic overview of the *in vitro* setup. The red line illustrates the inserted flexible microcatheter.

**Figure 3.**
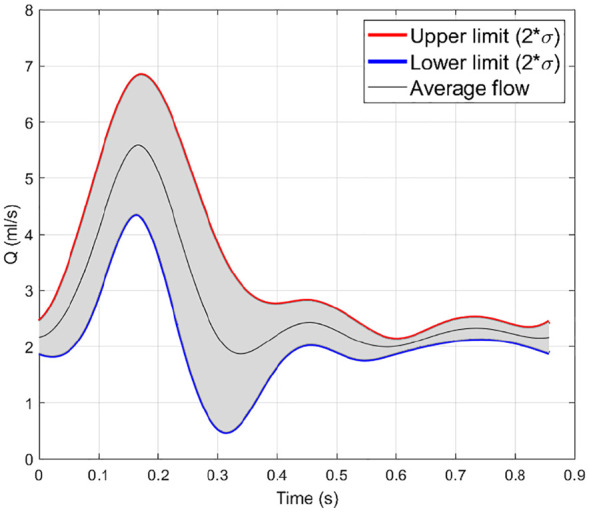
Measured flow profile in phantom.

A symmetrical phantom with circular successively bifurcating vessels was developed to study the behavior of the clinical catheter during microsphere injection. The model was 3D printed (Ultimaker S5, Ultimaker, Utrecht, The Netherlands) using acrylonitrile butadiene styrene (ABS). The model was then placed in a container and silicone Sylgard-184 (Dow Corning GmBH, Wiesbaden, Germany) was poured over the model to cast the phantom. After curing the silicone block, the phantom was flushed with acetone to remove the ABS, resulting in a cavity in the silicone block representing the lumen of the phantom. The phantom featured an inlet diameter comparable to the size of the right hepatic artery (RHA), 3.6 mm.^11^ Diameters after the first bifurcation were based on Murray’s law,^12^ and the angle of the first bifurcation was based on previously described models (Figure 1).^13^ As depicted in Figure 2, the phantom was incorporated in a flow circuit. The phantom inlet was connected to a 12 cm acrylic pipe to ensure fully developed flow at the inlet.^14^ The phantom outlets were uniform in length and did not include additional resistances. The outlets led to an open fluid collection reservoir, which was connected to a continuous pump (MGD1000P brushless micropump, RS Components Ltd., Corby, UK) and a pulsatile pump (SuperPump, ViVitro labs, Victoria, Canada). The RHA flow profile was set to 70 beats per minute (BPM), as proposed in the literature (Figure 3).^15^ Aramburu et al^15^ reported a mean velocity of 30 cm/s and systolic-peak velocity of 60 cm/s for the RHA, derived from a study by Hübner et al^16^ who performed Doppler sonography on 15 patients with various liver diseases. Considering an inlet diameter of 3.6 mm this translated to a mean flow of 3.1 mL/s and systolic-peak flow of 6.1 mL/s, which was simulated in the experiments by superimposing the continuous and pulsatile flows as the phantom’s inlet boundary condition.

The blood-mimicking fluid (BMF) in the experimental setup is a mixture of water, glycerol (98.0%–101.0% purity, VWR International, Radnor, USA), and urea (≥99.3% purity, Thermo Fisher Scientific Inc., Waltham, USA), with respective weight percentages of 55.8%, 34.2%, and 10%, yielding in a density of 1112 kg/m^3^ and viscosity of 3.5 cP.^17^ Before the start of an experiment, BMF was pumped via a flow sensor (Coriolis MX 55 flow sensor, Bronkhorst High-Tech B.V., Ruurlo, The Netherlands) directly to the collection reservoir (Figure 2), bypassing the phantom. Once the correct flow was established, collection vials were placed at the end of each phantom outlet and the BMF was directed through the phantom.

**Figure 4.**
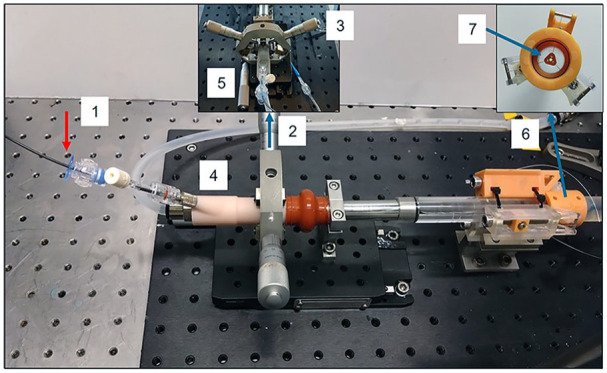
Design of the positioning device. The catheter was advanced at the red arrow (1). In the first part of the positioning device (2) 2 external screws (3) facilitated the radial steering of the 3D printed pipe enclosing the catheter (4). The longitudinal screw (5) allowed for axial adjustments. At the second part of the positioning device (6) the catheter passed through a small attachment (7). Here, 3 wires (0.30 mm diameter) were radially attached at 120° intervals. The wires were threaded through small openings in the inlet pipe and their length could be modified by tightening the attached screws. This approach was inspired by the positioning mount described by Richards et al.^18^ Distal to the second part of the positioning device enough length was left to enable full flow development before entrance of the phantom.

Microspheres were injected in the setup via a 130 cm 2.7Fr./0.90 mm outer diameter microcatheter (Progreat, Terumo Europe, Leuven, Belgium) with an inner diameter of 0.65 mm, enclosed by a straight-shaped 110 cm 5Fr./1.67 mm guiding catheter (Radifocus Glidecath, Terumo Europe, Leuven, Belgium). The distance from the guiding catheter to the first bifurcation in the phantom remained fixed at 11.5 cm. The microsphere injections were compared with injections via a stainless steel rigid catheter (outer diameter 0.90 mm, inner diameter 0.60 mm and length 40 cm), hereafter referred to as rigid catheter. The catheters were supported at 2 locations by a positioning device (Figure 4).

### Outflow Calibration

In a test run prior to the experiment, the flow created by the pulsatile and continuous pump ran through the phantom for 3 minutes and fluid at the outlets was collected. In this way it was checked whether the flow to all outlets was equal and influences by gravity were ruled out.

### Microsphere Injection

Non-radioactive holmium-165 loaded poly(l-lactic acid) microspheres (Quirem Medical B.V., Deventer, The Netherlands) with a mean size of 30 µm (±5 µm)^19^ and density of 1.4 g/mL^5^ were administered via a newly designed controlled administration device, which contains a rotating syringe to create a continuous microsphere suspension during administration.^20^ A 20 mL syringe was filled with 300 mg microspheres, 19 mL saline solution and 1 mL 0.1% phosphate buffered pluronic solution, leading to a microsphere suspension of 15 mg/mL (red syringe in Figure 2). With 2 syringe pumps, 1 from Harvard Apparatus (PHD ULTRA, Harvard Apparatus, Inc., USA) and 1 from New Era (NE-4000 Multi-Phaser, New Era Pump Systems Inc, USA) the administration protocol was kept as close as possible to clinical practice.^21^ Syringe pump 1 (Figure 2) administered the microsphere suspension with a pre-programmed pulsatile profile (pushes of 0.2 mL in 0.5s, followed by a 0.5s delay). Following the administration of 5 mL of the microsphere suspension, syringe pump 2 initiated a 5 mL flush using similar pump settings. This process was repeated 4 times until the microsphere suspension syringe was empty.

### Position and Motion of the Catheter

To study the motion of the clinical catheter, analysis was performed using 2 cameras with top and side view on the phantom, operating at 30 frames/second 3840 × 2160 image resolution (Logitech BRIO webcam, Logitech), resulting in approximately 135 pixels over the vessel lumen. The catheter was segmented from the images using thresholding. After image calibration the distance of the catheter to the upper phantom wall in all frames was calculated using custom MATLAB acquisition code (Matlab R2022a, MathWorks, Natick, Massachusetts). Subsequently, the angle recorded in the first video frame and the angle in the minimum and maximum catheter position with respect to the left phantom wall (top view camera) and upper phantom wall (side view camera) were calculated. The average angles and catheter positions were projected as an overlay on the camera output to position the rigid catheter at the average positions of the clinical catheter in subsequent experiments, to ensure identical placement.

A total of 45 measurements were performed, comprising 15 measurements using the clinical catheter and 30 measurements using the rigid catheter. For the rigid catheter, 2 separate sessions were made to position it at the location of the clinical catheter, resulting in 2 sets of 15 measurements each. Five repeated injections were performed at 3 distinct positions relative to the first bifurcation within the phantom: 0, 15, and 30 mm (Figure 1).

To visualize the behavior of the injection stream exiting the catheter, an extra experiment was performed in which the saline injection solution was mixed with a blue dye (Dr. Oetker, Bielefeld, Germany).

### Microsphere Distribution Analysis

Microspheres sedimented within 1 minute to the bottom of the collection vials, after which the supernatant (BMF and saline solution) was removed by suction with a pipette controller. Then, washing steps were performed with 30 mL distilled water and 0.1 mL ethanol 50% solution (v/v) to minimize surface tension. Following the initial wash, microspheres were suspended using a vortex and transferred into 50 mL centrifuge vials. The initial collection vials were rinsed with distilled water, and this residue was added to the centrifuge vials. After centrifugation (10 minutes, 1500 r/min), the supernatant was removed, and the washing step was repeated 2 more times. Subsequently, the centrifuge vials were placed in an oven at 40°C for 7 days to evaporate the distilled water. Weighing the vials allowed for the determination of the added microsphere weight; hence, the particle distribution among the outlets. Accuracy analysis for this weighing technique can be found in Supplemental 1.

For the catheter motion analysis and the microsphere distribution analysis, the Kolmogorov-Smirnov test (n≥50) and the Shapiro-Wilk test (n<50) were used, respectively, to determine whether the data were normally distributed. Mean and standard deviations (SDs) were used unless indicated otherwise. For comparison of the microsphere distribution between the clinical catheter and the rigid catheter the Pearson’s correlation coefficient (r) was used, with 0≤r<.1 none, .1≤r<.3 poor, .3≤r<.6 fair, .6 ≤ r<.8 moderate, 0.8≤r<1 very strong, r=1 perfect correlation.^22^ The data were processed using SPSS statistical software package version 26 (SPSS Inc. Chicago, IL, USA).

## Results

### Outflow Calibration

Fluid volume collected at the outlets during the calibration run showed maximum deviations of 1.2% (clinical catheter), 0.8% (rigid catheter set 1), and 1.0% (rigid catheter set 2) between the 8 outlets.

### Position and Motion of the Catheter

**Figure 5.**
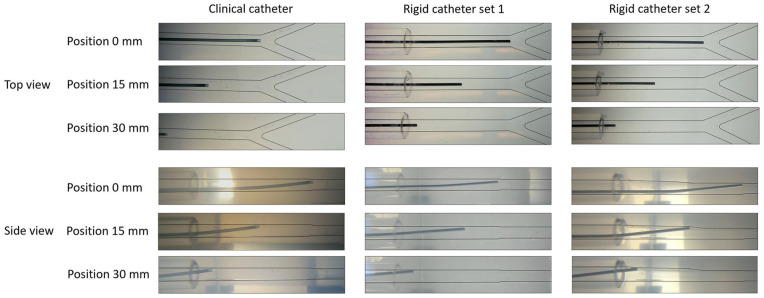
Position of the clinical and rigid catheter during the experiments.

**Figure 6.**
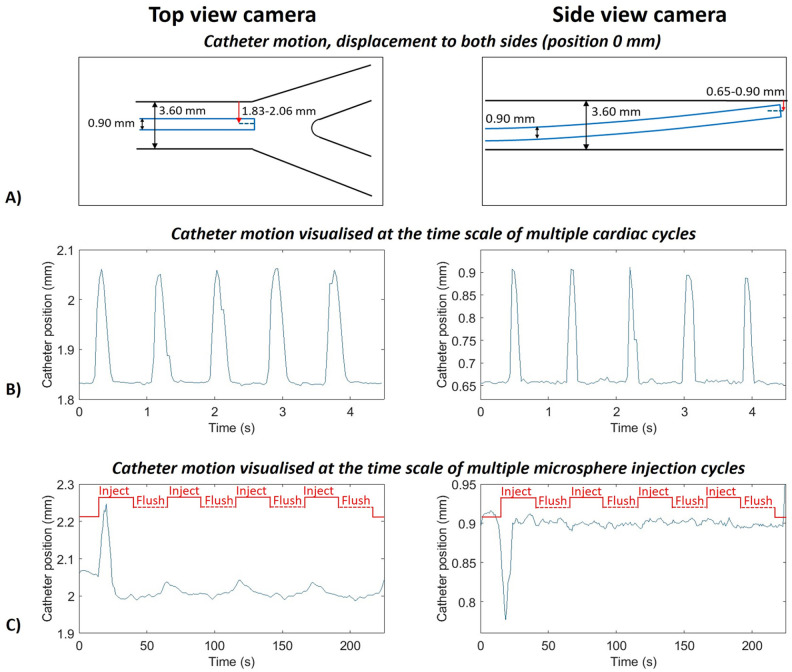
(A) Diagram of the motion of the clinical catheter, the red arrows show the distance of the middle of the catheter to the upper wall of the phantom. (B) Distance of the clinical catheter to the upper phantom wall (red arrow from A) for a period of 5 s for top view (left: 1.83 mm in rest position, 2.06 mm during peak systole) and side view (right: 0.65 mm in rest position, 0.90 mm during peak systole) to reveal the influence of the cardiac cycle on the motion of the catheter. (C) Peaks of the signal from figure B for a complete experiments (225 s) for top view (left) and side view (right), revealing the influence of the injection profile on the motion of the catheter. The microsphere injection cycle is annotated in red.

The true clinical catheter locations with respect to the start of the first bifurcation were: –0.31 mm (SD 0.05), 16.18 mm (SD 0.29), and 30.30 mm (SD 0.26), compared with the planned catheter positions 0, 15, and 30 mm (Figure 5). The clinical catheter, which had limited radial guidance, was positioned mostly against the upper vessel wall of the phantom (Figure 6A). Only during peak systole the catheter displayed motion away from the wall, as shown in Figure 6B. The catheter motion analysis revealed a repeated pattern in both the top view and side view camera videos, corresponding to the 70 BPM cardiac cycle (example in Figure 6B). Analysis of the complete injection profile revealed a second repeated pattern corresponding with the timepoints when microsphere injection was started (Figure 6C). During the first injection cycle some air bubbles were introduced, causing extra catheter motion, which is visible in the form of a prominent peak at the start of the first injection in Figure 6C. An example video of the motion of the catheter can be found in Supplemental 2.

The rigid catheter was steered to the mean position of the clinical catheter. First visual inspection (Figure 5) suggested equal placement of the 2 catheter types. However, in the detailed analysis of the top and side view videos it became clear that the angles did deviate from the clinical catheter orientation with the largest displacement for side view position 30 mm (Supplemental 3, 5.98° (SD 0.84) for the clinical catheter versus 4.02° (SD 0.08) for the rigid catheter). To improve the position of the clinical catheter in terms of angle and to eliminate any potential impact of an increased angle on the outflow distribution, a second set of experiments was obtained. In rigid catheter set 2, the angle for position 30 mm had a displacement of 6.16° (SD 0.09), a difference of 0.18° with respect to the clinical catheter in the side view. An overview of the position and angles of the catheters during the experiments is available in Supplemental 3.

The maximum displacement of the clinical and rigid catheter for each catheter position was 0.62, 0.52, and 0.87 mm, for position 0, 15, and 30 mm, respectively (Table 1), within a vessel of 3.6 mm. For the rigid catheter the maximum displacement was 0.07 mm.

**Table 1.** Maximum Displacement of the Catheter in Millimeters and in Percentages of Total Vessel Lumen Inside the Vessel Lumen for 3 Positions (0, 15, and 30 mm to the First Bifurcation in the Phantom).

Position	Maximum displacement in mm and in percentages of total vessel lumen					
	Clinical catheter		Rigid catheter set 1		Rigid catheter set 2	
	Top view	Side view	Top view	Side view	Top view	Side view
0 mm	0.51 (14.2%)	0.62 (17.2%)	0.04 (1.1%)	0.05 (1.4%)	0.02 (0.6%)	0.05 (1.4%)
15 mm	0.34 (9.4%)	0.52 (14.4%)	0.03 (0.8%)	0.07 (1.9%)	0.01 (0.3%)	0.06 (1.7%)
30 mm	0.32 (8.9%)	0.87 (24.2%)	0.01 (0.3%)	0.03 (0.8%)	0.03 (0.8%)	0.07 (1.9%)

Supplemental 4 illustrates that the stream exiting the catheter hit the wall of the phantom almost immediately after release from the catheter, due to the angle of the catheter. As a result, the injection stream spreads throughout the entire lumen, displaying a preference for the outer edges.

### Microsphere Distribution Analysis

**Figure 7.**
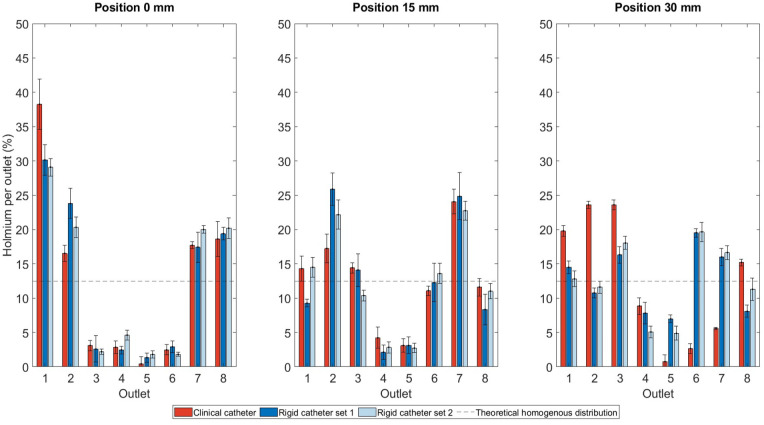
Amount of microspheres per outlet in percentages of the total of 8 outlets and standard deviation for 3 positions (0, 15, and 30 mm to the first bifurcation in the phantom). The black dashed line indicates a theoretical homogenous distribution among the outlets.

The microsphere distribution for the 3 planned positions (0, 15, and 30 mm upstream of the first bifurcation) can be found in Figure 7. At the 30 mm position, for the clinical catheter 75.9% of the microspheres went to the outlet 1–4, while for the rigid catheter the microspheres were more widely spread out over the phantom (49.4 and 47.6% to outlet 1–4 for rigid catheter set 1 and 2 respectively). For position 0 and 15 mm a very strong correlation in microsphere distributions between the clinical and rigid catheter per outlet can be observed. For rigid catheter set 1: r=.949, p<.001 (position 0 mm) and r=.899, p<.002 (position 15 mm). For rigid catheter set 2: r=.957, p<0.001 (position 0 mm) and r=.934, p<0.001 (position 15 mm). Position 30 mm shows no to poor correlation between the clinical and rigid catheter (for rigid catheter set 1: r=.047, p=0.911, for rigid catheter set 2: r=.190, p=0.653). The 2 experimental sets performed with the rigid catheter showed very strong correlation for all 3 positions (r>.900, p<0.001 for all 3 positions).

## Discussion

In TARE, achieving a favorable microsphere distribution (eg, high T/N ratios) is important. Improving the understanding of parameters that potentially influence the microsphere distribution could thus lead to improved patient treatment. Therefore, this study investigated the motion of a clinically used standard end-hole microcatheter during microsphere injection.

The most interesting finding from this study was the observed motion of the clinical catheter and its influence on the microsphere distribution. The results from position 30 mm are expected to be the most representative, as for this position the influence from the first bifurcation in the phantom was the smallest. For position 0 and 15 mm, the influence of the bifurcation cannot be ruled out and therefore the very strong correlation between the clinical and rigid catheter for position 0 and 15 mm (r>.899) should be interpreted with caution.^23^ Due to the pulsatile flow in the phantom, strengthened by the pulsatile microsphere injection, the clinical catheter showed maximum displacements of 0.87 mm, while for the rigid catheter the displacement was negligible (0.07 mm). This indicates that the observed motion in the clinical catheter is solely due to its flexibility, not to the design of the positioning device.

Particle release maps (PRMs) obtained via computer simulations have shown that microspheres injected in a specific radial section of the lumen exit at specific branches, since the majority of particles injected follow fluid streamlines.^13,23^ However, the PRMs change over the cardiac cycle, because the streamlines and their corresponding target outlets change at different time points in the cardiac cycle. In this vitro study each administration cycle lasted 25 seconds (5 mL pulsed injection, pushes of 0.2 mL in 0.5 s, followed by a 0.5 s delay) averaging several temporal particle injection conditions. This might explain why no precise targeting was found during this study. Another explanation could be that the microspheres were released at a 5° angle in the upper half of the lumen. Consequently, upon release from the catheter tip, the microspheres hit the wall of the phantom almost immediately, disrupting the injection stream exiting the catheter (Supplemental 4).

Although the catheter tip position is widely studied in computer simulations, these studies either did not incorporate a catheter in their simulations at all,^9,13^ or evaluated a starting position of a catheter and assume no catheter displacement during microsphere injection.^24–26^ However, the results from this study suggest that catheter motion does play an important role in microsphere distribution, especially further away from a bifurcation, and therefore simulating 1 starting position of the catheter may not be justified.

Computer simulations allow a degree of control over the (virtual) experimental setup which is impossible to achieve in an *in vitro* or *in vivo* study. Additionally, they may form the basis of computer-aided treatment planning tools as computer models can help to predict where and in what concentration microspheres will deposit. Therefore, it is important to rule out the influence of factors such as catheter motion, which is currently considered to be rigid in the computer simulations.

Earlier simulations also showed that small displacements in radial catheter location between consecutive sessions, most likely undetectable in current clinical practice, could lead to very different particle distributions.^9,13,27^ This contradicts our findings; the correlation between the 2 sets performed with the rigid catheter is strong, indicating that small displacements of up to 0.17 mm in position and 2.14° in angle would have minimal impact on the microsphere distribution. Possible explanations might be that the sensitivity of distributions to small changes is dependent on the radial and axial catheter position, or phantom related issues such as the specific geometry used in the different studies or the open outlets with no boundary conditions as chosen in this *in vitro* study.

During this study, the external blood flow was monitored continuously using a flow sensor and the catheter position was tracked during the complete injection using top and side view video cameras. Moreover, the combination of the clinical microcatheter with the guiding catheter, made the experiments more comparable to the clinical situation next to mimicking the pulsed injection scheme used in clinical practice. In the *in vitro* works up to now, either no catheter,^9^ a rigid catheter,^18,28^ or a nonguided microcatheter was used.^10,29–31^

The present study had several limitations. First, the silicone phantom had rigid vessel walls, a condition that differs from the *in vivo* scenario. Also, no boundary conditions were applied to the outlets of the phantom. Small changes in outlet boundary conditions can already change the microsphere distribution in the hepatic arterial tree. For example, Kennedy et al^32^ showed that a reduction of outlet pressure of 20% to a certain branch can reduce flow rate by 66% to this branch. However, in our model the main goal was to observe differences at the catheter tip solely due to motion of the catheter, without interference of other factors influencing the distribution. In future experiments, it might be useful to go toward more patient-specific geometries including varying outlet flows to see whether movement of the catheter still has an impact on the distribution. Another limitation is that only 1 clinically used standard end-hole microcatheter was studied (Progreat 2.7Fr.). Other microcatheters may exhibit varying degrees of motion. Aramburu et al^8^ investigated an angled tip and anti-reflux catheter and found important changes in microsphere distribution when injecting far from a bifurcation.

Potential errors in quantifying microspheres at each outlet may also arise. Despite flushing the setup between consecutive experiments, residual microspheres might be present in the setup, reaching outlets in subsequent experiments. Another source of error could be incomplete removal of glycerol during the washing protocol. Nevertheless, the low SDs in the repeated injections (Figure 7, SD<0.04), validate the replicability and mitigate the impact of introduced errors.

### Clinical Interpretation

In current clinical practice, planar angiographic images are acquired during the TARE procedure.^33^ From these images only the axial position of the catheter can be derived. This study has shown that the radial position of the catheter changes within the cardiac cycle and that this influenced the microsphere distribution. Kao et al^34^ also described this phenomenon *in vivo* in 3 case series; in the digital subtraction angiograms from 3 patients with unexpected microparticle distributions, it was found that the microcatheter was placed eccentrically with the tip abutting the arterial wall. This emphasizes the unpredictability of microsphere injections and highlights the importance for a more real-time prediction of microsphere distribution.

The most effective approach in current clinical practice for predicting microsphere distribution involves contrast injection under cone beam computed tomography (CBCT). CBCT contrast injections are performed continuously via a contrast media injector using injection rates up to 120 mL/min for vessel mapping. When the microcatheter is positioned at the intended treatment location, the primary limitation to maximum injection rate is the existence of reflux into nontarget branches.^35^ In contrast, actual microsphere injections are performed manually at significantly lower rates, typically between 18 and 36 mL/min.^31^ Our study identified increased motion of the catheter caused by the injection pattern (Figure 6C), which in turn influenced microsphere distribution (Figure 7). This suggests that contrast distribution may not accurately predict microsphere distribution. Therefore, aligning injection rates could improve prediction accuracy, by either reducing CBCT contrast injection rates or increasing microsphere injection rate. However, a higher microsphere injection rates is often undesirable, as high injection rates may result in the delivery of too many microspheres in the first few cycles of TARE. Another option could be to mix saline solution with contrast agent during the creation of a microsphere suspension. This could serve as a real-time predictor for microsphere distribution during angiography, as noted in the HEPAR trial.^36^

The same addresses for a technetium-99m-macroaggregated albumin (^99m^Tc-MAA) scout dose, as this injection technique also differs from the actual microsphere injections. Only the holmium-166 scout dose, which is administered with the same injection technique as the therapeutic dose, may provide a low variability in catheter motion. This might be contributing to the superior predictive accuracy of holmium-166 scout dose with respect to ^99m^Tc-MAA scout dose for microsphere distribution.^37^

Additionally, also during microsphere injections in TARE the specific injection technique can vary between institutes and interventional radiologists. The instructions for use for holmium microspheres recommends pulsatile injection at a rate of 0.1 mL per push per second.^21^ However, the speed of an individual push is not specified. The same applies to SIR-spheres (yttrium-90 resin microspheres), where slow pulsed injections of <5 mL/min are advised,^38^ and TheraSphere (yttrium-90 glass microspheres), where continuous injections ≥20 mL/min are advised.^39^ Future research should focus on the impact of different injection rates on the motion of the catheter tip and the corresponding microsphere distribution.

The current study focused exclusively on TARE. However, catheter motion may also have its impact on drug-eluting bead transarterial chemoembolization (DEB-TACE), as both procedures utilize microcatheters of similar sizes.^40^ Unlike TARE, where microspheres range from 25 to 32 µm,^5^ DEB-TACE particles are larger, with sizes between 100 and 700 µm.^40^ Microspheres tend to concentrate along the central axis of a blood vessel due to the wall exclusion effect.^41^ As a result, larger particles will follow a more centralized path within the vessel as the ratio of microsphere diameter to vessel diameter increases. Consequently, the behavior of DEB-TACE particles differs significantly and cannot be directly extrapolated from the findings of this study. Furthermore, the injection technique varies: holmium microspheres are delivered using a pulsed injection method, whereas DEB-TACE employs a slow, continuous injection approach.

For current clinical practice, it is important to maintain a consistent microsphere administration pattern, as this will minimize catheter motion variability and create a more predictable microsphere distribution pattern. A more predictive setting can also be achieved by performing injections further upstream from a bifurcation. This study demonstrated that injections performed further from a bifurcation allows microspheres to better align with the blood flow compared with injections administered closer to a bifurcation, as the microspheres distributed more evenly over the 8 outlets for the 30 mm distance from the bifurcation (Figure 7). Taebi et al^23^ also reported that the impact of radial catheter motion had a weaker effect on microsphere distribution when the injection plane was further away from the bifurcation (20 mm).

## Conclusion

This *in vitro* study has shown that both the pulsatile flow in the *in vitro* model and the pulsatile injection of microspheres cause radial motion of a clinical microcatheter. Inside the phantom’s vessel lumen of 3.6 mm, displacements up to 0.87 mm (24.2%) were observed, affecting the distribution of microspheres especially at 30 mm distance from the first bifurcation in the phantom. Since catheter motion has an impact on microsphere distribution, a lower variability in microsphere administration pattern will create a more consistent catheter motion and may therefore facilitate a more predictable microsphere distribution pattern. Moreover, based on our data, administering as far as possible from a bifurcation will improve alignment of the microspheres with the blood flow, which will improve targeting toward hypervascular tumor sites. Future research is required to assess the true clinical implications and will help to determine whether administration guidelines should be further optimized in order to minimize interprocedural differences.

## Supplemental Material

sj-docx-1-jet-10.1177_15266028251318953 – Supplemental material for In Vitro Investigation of Microcatheter Behavior During Microsphere Injection in Transarterial Radioembolization Supplemental material, sj-docx-1-jet-10.1177_15266028251318953 for In Vitro Investigation of Microcatheter Behavior During Microsphere Injection in Transarterial Radioembolization by Tess Josien Snoeijink, Jan Lucas van der Hoek, Hadi Mirgolbabaee, Tristan Gerard Vlogman, Joey Roosen, Johannes Frank Wilhelmus Nijsen and Erik Groot Jebbink in Journal of Endovascular Therapy

sj-docx-2-jet-10.1177_15266028251318953 – Supplemental material for In Vitro Investigation of Microcatheter Behavior During Microsphere Injection in Transarterial Radioembolization Supplemental material, sj-docx-2-jet-10.1177_15266028251318953 for In Vitro Investigation of Microcatheter Behavior During Microsphere Injection in Transarterial Radioembolization by Tess Josien Snoeijink, Jan Lucas van der Hoek, Hadi Mirgolbabaee, Tristan Gerard Vlogman, Joey Roosen, Johannes Frank Wilhelmus Nijsen and Erik Groot Jebbink in Journal of Endovascular Therapy

sj-docx-3-jet-10.1177_15266028251318953 – Supplemental material for In Vitro Investigation of Microcatheter Behavior During Microsphere Injection in Transarterial Radioembolization Supplemental material, sj-docx-3-jet-10.1177_15266028251318953 for In Vitro Investigation of Microcatheter Behavior During Microsphere Injection in Transarterial Radioembolization by Tess Josien Snoeijink, Jan Lucas van der Hoek, Hadi Mirgolbabaee, Tristan Gerard Vlogman, Joey Roosen, Johannes Frank Wilhelmus Nijsen and Erik Groot Jebbink in Journal of Endovascular Therapy

sj-docx-4-jet-10.1177_15266028251318953 – Supplemental material for In Vitro Investigation of Microcatheter Behavior During Microsphere Injection in Transarterial Radioembolization Supplemental material, sj-docx-4-jet-10.1177_15266028251318953 for In Vitro Investigation of Microcatheter Behavior During Microsphere Injection in Transarterial Radioembolization by Tess Josien Snoeijink, Jan Lucas van der Hoek, Hadi Mirgolbabaee, Tristan Gerard Vlogman, Joey Roosen, Johannes Frank Wilhelmus Nijsen and Erik Groot Jebbink in Journal of Endovascular Therapy

sj-docx-5-jet-10.1177_15266028251318953 – Supplemental material for In Vitro Investigation of Microcatheter Behavior During Microsphere Injection in Transarterial Radioembolization Supplemental material, sj-docx-5-jet-10.1177_15266028251318953 for In Vitro Investigation of Microcatheter Behavior During Microsphere Injection in Transarterial Radioembolization by Tess Josien Snoeijink, Jan Lucas van der Hoek, Hadi Mirgolbabaee, Tristan Gerard Vlogman, Joey Roosen, Johannes Frank Wilhelmus Nijsen and Erik Groot Jebbink in Journal of Endovascular Therapy

sj-tif-6-jet-10.1177_15266028251318953 – Supplemental material for In Vitro Investigation of Microcatheter Behavior During Microsphere Injection in Transarterial Radioembolization Supplemental material, sj-tif-6-jet-10.1177_15266028251318953 for In Vitro Investigation of Microcatheter Behavior During Microsphere Injection in Transarterial Radioembolization by Tess Josien Snoeijink, Jan Lucas van der Hoek, Hadi Mirgolbabaee, Tristan Gerard Vlogman, Joey Roosen, Johannes Frank Wilhelmus Nijsen and Erik Groot Jebbink in Journal of Endovascular Therapy
