# Supplementary material for: In Vitro Investigation of Microcatheter Behavior During Microsphere Injection in Transarterial Radioembolization
Source: J Endovasc Ther. 2025 Feb 24;33(4):1783–93. doi: 10.1177/15266028251318953 (PMC13371155; doi:10.1177/15266028251318953)
Supplement: sj-docx-1-jet-10.1177_15266028251318953 – Supplemental material for In Vitro Investigation of Microcatheter Behavior During Microsphere Injection in Transarterial Radioembolization [file sj-docx-1-jet-10.1177_15266028251318953.docx]

Table E1: Validation test 1, ten known amounts of holmium-165 microspheres were brought into suspension with blood-mimicking fluid (composed of water, glycerol and urea) and saline solution and washed three times.

| **Test** | **Weighed amount of microspheres (mg)** | **Recovered amount of microspheres (mg)** | **Deviation (mg)** |
| --- | --- | --- | --- |
| 1 | 5.8 | 5.5 | -0.3 |
| 2 | 9.9 | 14.3 | 4.4 |
| 3 | 24.9 | 27.7 | 2.8 |
| 4 | 36.4 | 39.1 | 2.7 |
| 5 | 45.3 | 50.2 | 4.9 |
| 6 | 56.6 | 61.1 | 4.5 |
| 7 | 74.5 | 80.3 | 5.8 |
| 8 | 103.1 | 108.8 | 5.7 |
| 9 | 127.4 | 132.6 | 5.2 |
| 10 | 151.4 | 155.6 | 4.2 |
