# Supplementary material for: In Vitro Investigation of Microcatheter Behavior During Microsphere Injection in Transarterial Radioembolization
Source: J Endovasc Ther. 2025 Feb 24;33(4):1783–93. doi: 10.1177/15266028251318953 (PMC13371155; doi:10.1177/15266028251318953)
Supplement: sj-docx-2-jet-10.1177_15266028251318953 – Supplemental material for In Vitro Investigation of Microcatheter Behavior During Microsphere Injection in Transarterial Radioembolization [file sj-docx-2-jet-10.1177_15266028251318953.docx]

Table E2: Validation test 2, three known amounts of holmium-165 microspheres were brought into suspension with blood-mimicking fluid (composed of water, glycerol and urea) and saline solution and washed three times with centrifuge steps in between.

| **Test** | **Weighed amount of microspheres (mg)** | **Recovered amount of microspheres (mg)** | **Deviation (mg)** |
| --- | --- | --- | --- |
| 1 | 48.9 | 50.1 | 1.2 |
| 2 | 49.1 | 51.2 | 2.1 |
| 3 | 57.1 | 58.3 | 1.2 |
