# Supplementary material for: In Vitro Investigation of Microcatheter Behavior During Microsphere Injection in Transarterial Radioembolization
Source: J Endovasc Ther. 2025 Feb 24;33(4):1783–93. doi: 10.1177/15266028251318953 (PMC13371155; doi:10.1177/15266028251318953)
Supplement: sj-docx-3-jet-10.1177_15266028251318953 – Supplemental material for In Vitro Investigation of Microcatheter Behavior During Microsphere Injection in Transarterial Radioembolization [file sj-docx-3-jet-10.1177_15266028251318953.docx]

Table E3: Catheter distance to the upper wall of the phantom, angle of the catheter, and distance to the bifurcation for the clinical catheter and the two sets performed with the rigid catheter, calculated in the top view videos.

| **Position** | **Distance to upper wall phantom (mm)** | | | **Angle (degrees)** | | | **Distance to bifurcation (mm)** | | |
| --- | --- | --- | --- | --- | --- | --- | --- | --- | --- |
|  | *Clinical* | *Rigid* | *Rigid 2* | *Clinical* | *Rigid* | *Rigid 2* | *Clinical* | *Rigid* | *Rigid 2* |
|  | *Median (IQR)^a^* | *Median (IQR)* | *Median (IQR)* | *Mean (SD)^b^* | *Mean (SD)* | *Mean (SD)* | *Mean* | *Mean* | *Mean* |
| 0 mm | 1.84 (0.07) | 1.93 (0.01) | 1.82 (0.06) | -0.77 (0.23) | -0.22 (0.14) | 0.56 (1.0) | -0.31 | 0.24 | -0.22 |
| 15 mm | 2.15 (0.10) | 2.03 (0.02) | 1.86 (0.05) | -0.91 (0.27) | -0.02 (0.07) | 0.06 (0.07) | 16.18 | 16.39 | 15.74 |
| 30 mm | 1.71 (0.10) | 1.68 (0.01) | 1.65 (0.07) | -0.79 (0.37) | 0.02 (0.04) | -0.03 (0.06) | 30.30 | 29.75 | 30.27 |

^a^ IQR = Interquartile range
^b^ SD = Standard deviation
