# Supplementary material for: In Vitro Investigation of Microcatheter Behavior During Microsphere Injection in Transarterial Radioembolization
Source: J Endovasc Ther. 2025 Feb 24;33(4):1783–93. doi: 10.1177/15266028251318953 (PMC13371155; doi:10.1177/15266028251318953)
Supplement: sj-docx-4-jet-10.1177_15266028251318953 – Supplemental material for In Vitro Investigation of Microcatheter Behavior During Microsphere Injection in Transarterial Radioembolization [file sj-docx-4-jet-10.1177_15266028251318953.docx]

Table E4: Catheter distance to the upper wall of the phantom and angle of the catheter, for the clinical catheter and the two set performed with the rigid catheter, calculated in the side view videos.

| **Position** | **Distance to upper wall phantom (mm)** | | | **Angle (degrees)** | | |
| --- | --- | --- | --- | --- | --- | --- |
|  | *Clinical* | *Rigid* | *Rigid 2* | *Clinical* | *Rigid* | *Rigid 2* |
|  | *Median (IQR) ^a^* | *Median (IQR)* | *Median (IQR)* | *Mean (SD) ^b^* | *Mean (SD)* | *Mean (SD)* |
| 0 mm | 0.63 (0.14) | 0.47 (0.03) | 0.49 (0.03) | 4.93 (0.95) | 3.56 (0.08) | 4.81 (0.39) |
| 15 mm | 0.40 (0.19) | 0.45 (0.02) | 0.38 (0.05) | 4.98 (0.98) | 3.86 (0.10) | 5.59 (0.10) |
| 30 mm | 0.35 (0.11) | 0.45 (0.02) | 0.44 (0.02) | 5.98 (0.84) | 4.02 (0.08) | 6.16 (0.09) |

^a^ IQR = Interquartile range
^b^ SD = Standard deviation
