# Supplementary material for: In Vitro Investigation of Microcatheter Behavior During Microsphere Injection in Transarterial Radioembolization
Source: J Endovasc Ther. 2025 Feb 24;33(4):1783–93. doi: 10.1177/15266028251318953 (PMC13371155; doi:10.1177/15266028251318953)
Supplement: sj-docx-5-jet-10.1177_15266028251318953 – Supplemental material for In Vitro Investigation of Microcatheter Behavior During Microsphere Injection in Transarterial Radioembolization [file sj-docx-5-jet-10.1177_15266028251318953.docx]

**Supplemental file 1 Microsphere concentration analysis**

To determine the amount of microspheres collected at the outlets, a wash and dry method was developed and validated to obtain the dry weight of the microspheres. In a first validation test, ten known amounts of non-irradiated holmium-165 loaded microspheres were washed in the original collection vials. The microspheres sedimented to the bottom, the supernatant (BMF + saline solution) was removed and the microspheres were washed three times with 20 ml of distilled water and 0.1 ml ethanol 50% (v/v). After evaporating the residual fluid in an oven at 40 degrees, all deviations were below 5.8 mg (Table E1).

During this first validation test it was experienced that some of the microspheres did not sediment, even after reducing the surface tension with ethanol 50% (v/v). Therefore, it was decided that after the first washing step the suspension should be poured into 50 ml centrifuge vials. The original collection vials were rinsed with distilled water and all fluid was added to the new centrifuge vials. The microspheres were then centrifuged (10 minutes, 1500 rpm) before removal of the supernatant and the two remaining washing steps, alternated with a centrifuging step, were executed. This was tested for three samples, all deviations were below 2.1 mg (Table E2).

**Supplemental file 2**

Example video of the motion of the catheter from the top view camera and side view camera can be found in Figure E1 and E2 respectively.

**Supplemental file 3**

Catheter position for the clinical catheter and the rigid catheter set 1 and 2 can be found in Table E3 and E4. The distance to the upper wall of the phantom is provided, as well as the angle of the catheter and the axial distance towards the first bifurcation in the phantom.

**Supplemental file 4**

In Fig. E3 the result from an injection of saline solution mixed with a blue dye is provided, to visualize the behaviour of the injection stream exiting the catheter. The injected fluid hits the wall of the phantom almost immediately after release from the catheter, due to the angle of the catheter (visible in the side view). As a result, the injection stream spreads throughout the entire lumen, favouring the outer edges.
