# Supplementary figures and images for: In Vitro Investigation of Microcatheter Behavior During Microsphere Injection in Transarterial Radioembolization
Source: J Endovasc Ther. 2025 Feb 24;33(4):1783–93. doi: 10.1177/15266028251318953 (PMC13371155; doi:10.1177/15266028251318953)

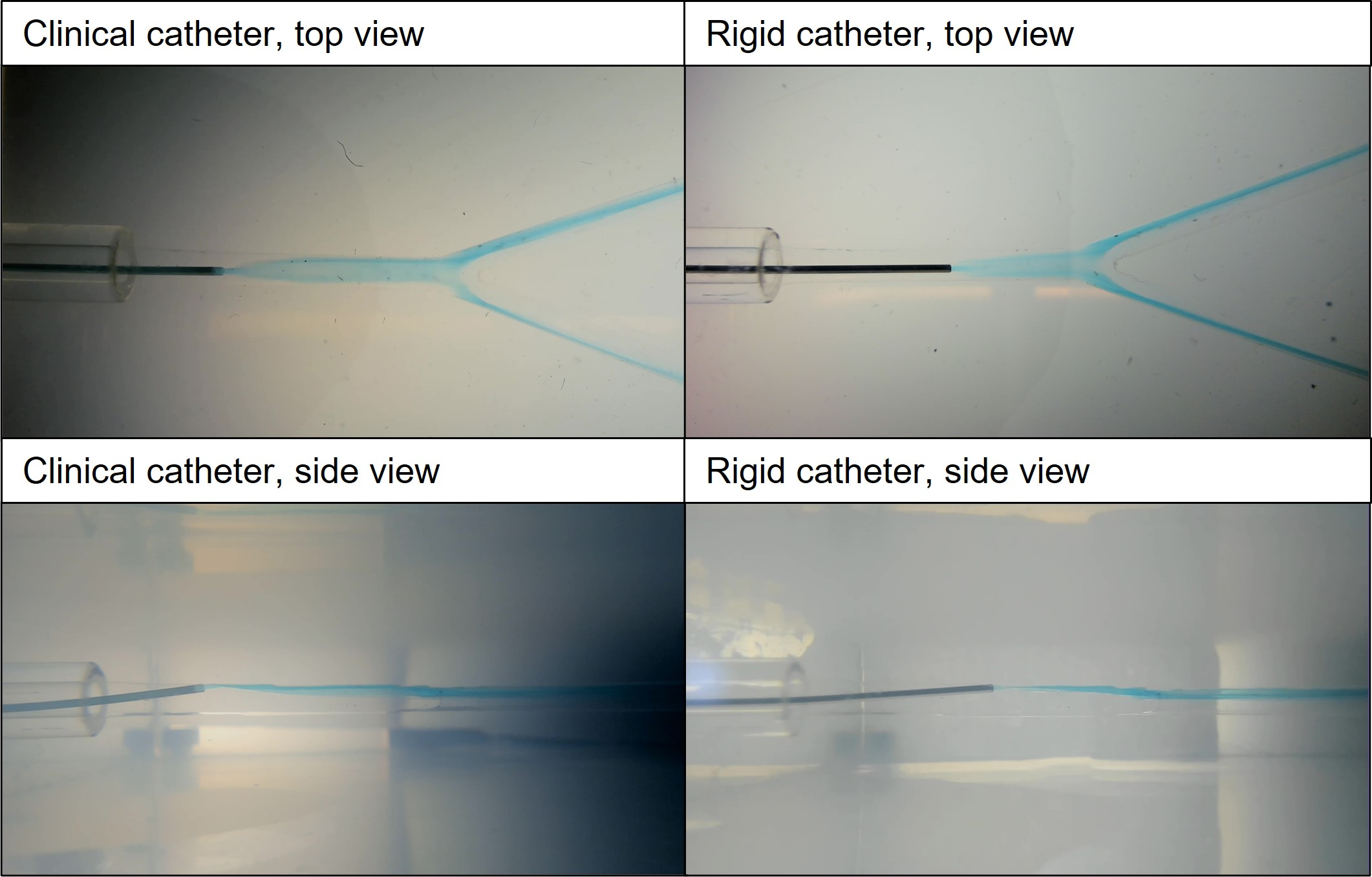

Supplement: sj-tif-6-jet-10.1177_15266028251318953 – Supplemental material for In Vitro Investigation of Microcatheter Behavior During Microsphere Injection in Transarterial Radioembolization [file sj-tif-6-jet-10.1177_15266028251318953.tif]
